# The Role of PET Tracers in Small-Cell Prostate Cancer (SCPC): An Overview in Clinical and Preclinical Settings

**DOI:** 10.3390/cancers18101645

**Published:** 2026-05-20

**Authors:** Flaminia Vocaturo, Silvia Taralli, Valentina Scolozzi, Lucia Leccisotti, Carmelo Caldarella

**Affiliations:** 1 Unit of Nuclear Medicine, Department of Diagnostic Imaging and Radiation Oncology, Fondazione Policlinico Universitario A. Gemelli IRCCS, L.go Francesco Vito, 1, 00168 Rome, Italy; flaminia.vocaturo01@icatt.it (F.V.); silvia.taralli@policlinicogemelli.it (S.T.); lucia.leccisotti@policlinicogemelli.it (L.L.); carmelo.caldarella@policlinicogemelli.it (C.C.); 2 Section of Nuclear Medicine, Department of Radiological Sciences and Hematology, Università Cattolica del Sacro Cuore, L.go Francesco Vito, 1, 00168 Rome, Italy

**Keywords:** small-cell prostate cancer, SCPC, PET/CT, neuroendocrine prostate cancer, molecular imaging

## Abstract

Background/Objectives: Small-cell prostate cancer (SCPC) is a rare, aggressive variant of prostate cancer with poor prognosis, arising “de novo” or through lineage plasticity from conventional adenocarcinoma under androgen receptor-targeted therapies. Characterized by low PSA levels despite high tumor burden and visceral metastases, SCPC poses diagnostic challenges with conventional and PSMA-targeted imaging due to variable tracer uptake. This narrative review aims to evaluate the role of PET/CT tracers in clinical and preclinical settings for SCPC diagnosis, staging, and management. Methods: A systematic literature search was conducted on PubMed and Scopus up to December 2025 using terms “PET OR positron emission tomography AND prostate OR prostatic AND small-cell NOT non-small-cell”. Eight studies (five clinical, three preclinical) on the role of PET/CT imaging in SCPC were included and analyzed for study design, population, tracers, and findings, with comparative evaluation of diagnostic performance across PET tracers. Results: Clinical studies showed that ^11^C-choline detects progression at low PSA but misses SCPC; ^18^F-FDG exhibited a high SUVmax value for distinguishing SCPC from adenocarcinomas with neuroendocrine differentiation, predicting poor survival; ^68^Ga-DOTATATE identified NEPC/SCPC with promising prognostic/therapeutic value for selected cases. Preclinical models evaluated ^89^Zr-tracers targeting DLL3 or CDCP1 (an antigen expressed in aggressive neuroendocrine tumours) and ^18^F-BnTP (a target of mitochondrial activity) in SCPC subtypes, focusing on translational imaging. Conclusions: From this review, although still based on limited literature evidence and mostly derived from retrospective and small SCPC sub-cohorts,^18^F-FDG PET/CT currently appears as the most reliable tracer for SCPC, aiding tumor detection and prognostication when PSMA/choline imaging fails. In the preclinical setting, DLL3/CDCP1-targeted agents emerge as promising theranostics tools. Multimodal imaging approach and prospective trials are needed for standardization and patient-based SCPC management.

## Simple Summary

Small-cell prostate cancer (SCPC) is a rare, aggressive variant of prostate cancer with poor prognosis, and poses diagnostic challenges with conventional and PSMA-targeted imaging due to variable tracer uptake. This narrative review summarizes the role of PET/CT tracers in clinical and preclinical settings for SCPC diagnosis, staging, and management. From our research, ^18^F-FDG PET/CT seems to be the most reliable tracer for SCPC, aiding detection and prognostication in cases when PSMA or choline PET/CT fails; emerging DLL3/CDCP1-targeted agents promise theranostics.

## 1. Introduction

Small-cell prostate cancer (SCPC) represents a rare and highly aggressive variant of prostate cancer, carrying a poor prognosis. It accounts for approximately 1% of all primary prostate cancers at initial presentation (the so-called de novo SCPC); however, its prevalence is substantially higher (10–20%) in advanced cases and, particularly, in metastatic castration-resistant prostate cancer (mCRPC), where SCPC often emerges after androgen receptor-targeted therapies due to treatment-induced neuroendocrine trans-differentiation [1,2,3,4]. In these latter cases, SCPC arises via trans-differentiation of conventional prostate adenocarcinoma under the selective pressure of androgen receptor-targeted therapies, like enzalutamide and abiraterone [5,6], a process known as lineage plasticity which leads to the development of a neuroendocrine phenotype with reduced androgen receptor (AR) signalling and marked neuroendocrine marker expression [1,7,8,9]. Histologically, SCPC may be seen as a pure small-cell entity or intermixed with typical prostate adenocarcinoma; both components usually share molecular alterations, supporting a common clonal origin [10,11].

From a clinical point of view, SCPC usually undergoes rapid disease progression, with early metastatic spread to visceral organs and soft tissues, and tumour burden is often disproportionately high relative to serum PSA levels [2]: most SCPC patients present with locally advanced or metastatic disease, typically exhibiting low or only mild elevated PSA, despite extensive tumour involvement [12,13]. Unlike conventional prostate cancers, SCPC is largely unresponsive to hormonal therapy and is, instead, managed using platinum-based chemotherapy regimens, paralleling the approach used for small-cell lung carcinoma [2,12,13,14]. The prognosis remains poor, with a median overall survival in metastatic disease shorter than 12 months [6].

Accurate staging and disease characterization are critical for guiding SCPC management. In this regard, conventional imaging modalities such as CT, MRI, and bone scintigraphy have significant limitations in detecting nodal and distant metastases, particularly in such neuroendocrine and small-cell subtypes characterized by small-size lesions, usually with non-significant changes on structural imaging [15,16].

Positron emission tomography/computed tomography (PET/CT) has an established role in the evaluation of advanced prostate cancer, leveraging a range of radiotracers to interrogate distinct molecular and metabolic features of disease. The American Society of Clinical Oncology and the European Society of Urology recommend PET/CT with various radiopharmaceuticals for advanced prostate cancer, noting the superior diagnostic performance (and theranostic advantages) of prostate-specific membrane antigen (PSMA)-targeted agents in most settings; however, their utility in SCPC remains uncertain due to variable PSMA expression [16,17,18]. Conversely, fluorine-18 fluorodeoxyglucose (^18^F-FDG) PET/CT, which reflects glucose metabolism, may have increased sensitivity in aggressive, dedifferentiated, or neuroendocrine tumours, including SCPC, often showing low PSMA expression and high glycolytic activity [19,20].

The current literature on PET/CT in SCPC is limited, with few studies directly comparing the performance of different tracers. Available data suggest that ^18^F-FDG PET/CT may offer advantages in clinical staging and assessment of tumour biology, while somatostatin analogues and other emerging tracers could provide additional insight on theranostic applications [20,21]. Further research is needed to establish standardized imaging protocols and clarify the prognostic and therapeutic implications of PET/CT findings in SCPC.

Our narrative review aims to summarize and analyze recent evidence on the role of PET/CT imaging in diagnosing, staging, and managing SCPC, providing an overview of the literature in such a rare disease.

## 2. Materials and Methods

**Figure 1:**
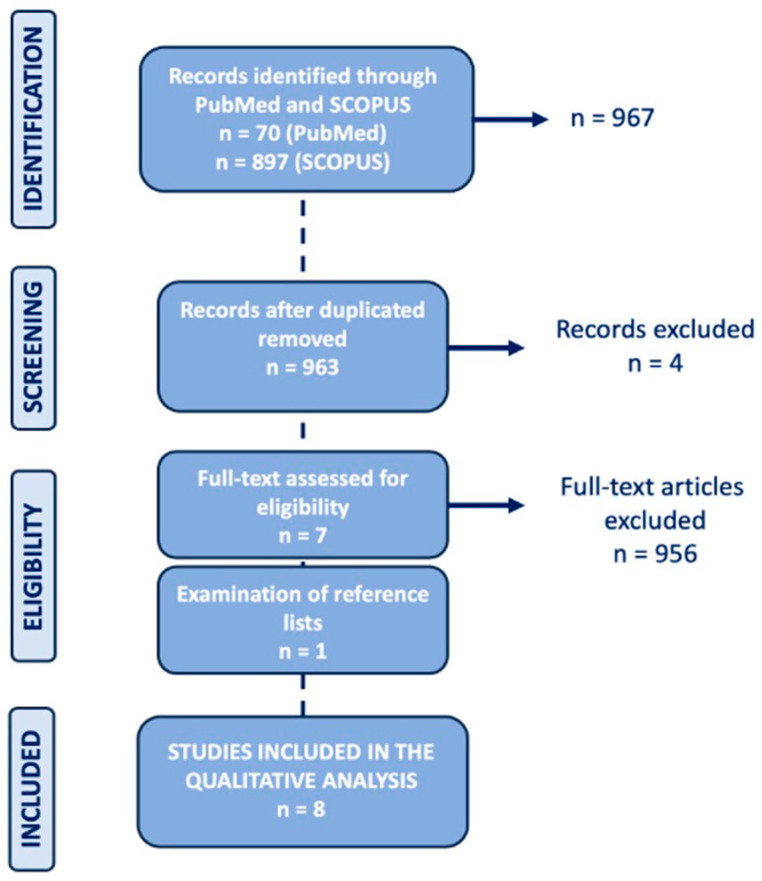
Flowchart of the literature selection.

A comprehensive literature search was conducted using the PubMed and Scopus databases (updated as of December 2025), employing the following search string: “(PET OR positron emission tomography) AND (prostate OR prostatic) AND small-cell NOT (non-small-cell)”. To expand our search, references of the retrieved articles were also screened for additional studies. Only clinical and preclinical studies providing data on the role of PET/CT imaging in SCPC, written in the English language, were considered potentially eligible for inclusion. Exclusion criteria included reviews, case reports, and studies unrelated to PET/CT imaging in SCPC. Titles and abstracts of the retrieved studies were screened independently by two researchers (FV and CC). The same two researchers then independently reviewed the full-text version of the selected articles for final eligibility. From PubMed, 70 papers were initially retrieved. Similarly, from Scopus, 897 documents were identified, four of which overlapped with the PubMed search. After removal of duplicates, screening of titles and abstracts and subsequent full-text analysis, 7 articles were considered eligible; an additional eligible article was identified after reference list analysis. So, eight articles were finally included in this review [22,23,24,25,26,27,28,29]. The research flowchart is presented in Figure 1.

For each included article, information was collected about the main study characteristics (study design and objectives, study population characteristics, PET technical aspects, study limitations) and (when available) on possible future perspectives. A comparative analysis among included studies was also performed to critically evaluate applicability, diagnostic performance, and clinical implications of each PET tracer, specifically for SCPC.

## 3. Results

Among the eight included studies, five were clinical and three were preclinical investigations on animal models; all were published in the period 2020–2024. Clinical studies were all retrospectively designed and one of them (Zhao et al.) [27] included an additional preclinical analysis. The three preclinical studies included both in vivo and in vitro analysis. The main characteristics of the included studies are reported in Table 1.

### 3.1. Clinical Studies

Across the five clinical studies, none focused exclusively on SCPC. Rather, SCPC or no-otherwise-specified neuroendocrine prostate cancer (NEPC) cases represented a minority subpopulation, within broader patients with metastatic or recurrent prostate adenocarcinomas.

Particularly, in the study from Mahmoud et al. [22], from an initial cohort of 1323 patients with radiographic disease progression (rDP) detected on ^11^C-choline PET/CT, 220 individuals with low PSA (<0.5 ng/mL) were selected. At the time of initial diagnosis, 6 patients out of 220 (3%) with PSA < 0.5 ng/mL were identified as having small cell-neuroendocrine prostate cancer (SC-NEPC); subsequently, at the time of rDP, 55/220 patients (25%) underwent repeat biopsy, of which 42/55 yielded evaluable samples: final histology revealed adenocarcinoma in 33 patients (79%) and SC-NEPC in 9 patients (21%). Mahmoud et al. [22] conducted a retrospective observational study on a real-world cohort from a single institution using registry data. They analyzed radiographic disease progression (rDP) in prostate cancer patients with very low PSA levels (<0.5 ng/mL) using ^11^C-choline PET/CT. Out of 1323 patients, 220 (16.6%) showed disease progression on ^11^C-choline PET despite low or undetectable PSA. Among a subpopulation biopsied at progression, approximately 21% exhibited small cell/neuroendocrine features, and the risk of having an unfavourable histology as SC-NEPC was much higher in patients with rDP at very low PSA levels than in patients with rDP at higher PSA levels. These findings emphasize that low PSA does not exclude aggressive disease progression, since undifferentiated histological subtypes may not be able to increase the laboratory marker despite an increase in tumour burden. PET/CT imaging can detect biologically aggressive forms like SCPC/NEPC before symptoms or conventional imaging methods reveal them. In another retrospective study by Shen et al. [23], a cohort of 44 patients with NEPC, including 13 small-cell neuroendocrine carcinoma (SCNC) and 31 adenocarcinomas with neuroendocrine differentiation (Ad-NED), was analyzed. The authors [23] conducted a retrospective multicentre study assessing the role of ^18^F-FDG PET/CT in the diagnosis and prognosis of neuroendocrine prostate cancer (NEPC), with a focus on SCNC. The study aimed to evaluate the association between FDG uptake degree, expressed as the Maximum Standardized Uptake Value (SUVmax), and histologic subtypes dichotomized as SCNC vs. Ad-NED, overall survival, and progression-free survival. Forty-four NEPC patients (13 SCNC and 31 Ad-NED) underwent ^18^F-FDG PET/CT before biopsy: the results showed significantly higher SUVmax in SCNC compared to Ad-NED (15.0 vs. 6.7; *p* < 0.0001). SUVmax cut-off >10.2 demonstrated good diagnostic accuracy to distinguish SCNC from Ad-NED (AUC 0.88). Both SCNC histology and SUVmax > 10.2 were associated with worse overall survival. Therefore, ^18^F-FDG PET/CT imaging may non-invasively identify SCNC among NEPC subtypes due to the higher glucose uptake, and elevated SUVmax serves as a predictor of poor prognosis, suggesting PET/CT may aid diagnosis and risk stratification in NEPC patients.

In the study by Zhao et al. [27], among 119 biopsies from patients with mCRPC and 12 patient-derived xenografts (PDXs), both SCNC and adenocarcinoma histology were represented, although the proportion of SCNC cases was not specified.

Telo et al. [28] evaluated a large series of ^11^C-choline PET/CT scans in 5792 patients with biochemical recurrence of prostate cancer at high PSA levels. The study focused on PET/CT negativity in patients with biochemical recurrence (BCR) and PSA levels above 20 ng/mL. While ^11^C-choline PET generally shows high sensitivity in this setting, some patients still exhibited negative scans. Although the exact proportion of SCNC within this cohort was not reported, the authors [28] mentioned SCPC as a possible biological explanation for false-negative results on ^11^C-choline PET/CT, highlighting that neuroendocrine prostate carcinomas, such as SCPC, may evade tracer uptake.

Last, Bilen et al. [29] conducted a retrospective study on 16 patients with metastatic castration-resistant prostate cancer (mCRPC), including two confirmed cases of neuroendocrine prostate cancer (NePC), one of them involving a mixed small-cell neuroendocrine carcinoma and acinar adenocarcinoma. The study evaluated the prognostic utility of ^68^Ga-DOTATATE PET/CT in this clinical setting. All patients showed tracer-avid lesions, with the two NePC cases, including the small cell form, exhibiting high tracer uptake (SUVmax > liver background) also being associated with poor prognosis. Therefore, DOTATATE PET/CT may be a valuable tool for early identification of neuroendocrine differentiation, including SCPC, and for risk stratification in mCRPC patients.

Methodological details about PET/CT acquisition available from clinical studies are summarized in Table 2.

### 3.2. Preclinical Studies

All three exclusively preclinical investigations focused on translational imaging but, similarly to the above reported clinical studies, did not limit their analyses exclusively to SCPC. More in detail, preclinical studies by Chou et al. [24] and Sharma et al. [25] have evaluated the diagnostic efficacy and potential therapeutic role of delta-like ligand 3 (DLL3)-targeted radiotracers, with DLL3 being an antigen expressed in aggressive neuroendocrine tumours, including small-cell lung cancer and neuroendocrine prostate cancer, and serves as an emerging target for antibody-based therapies [24].

Particularly, in the study by Chou et al. [24], 24 biopsies obtained from patients with mCRPC were examined, equally distributed between SCPC (*n* = 12) and adenocarcinoma (*n* = 12): imaging and binding studies on histological samples were performed using DLL3-targeted radiopharmaceuticals. They developed a novel PET radiopharmaceutical, [^89^Zr]DFO-DLL3-scFv, which specifically binds DLL3 via a single-chain variable fragment (scFv) labelled with Zirconium-89 (^89^Zr). In murine PDX models, the tracer demonstrated high sensitivity and specificity for DLL3-positive tumours and distinguished tumours with high, low, or absent DLL3 expression. The study also evaluated AMG 757, a DLL3-targeting bispecific T-cell engager, showing anti-tumour efficacy in SCNC animal models with varying DLL3 expression. Additionally, a single clinical case of SCNC treated with AMG 757 showed a confirmed partial response per RECIST criteria, within an ongoing clinical trial (NCT04702737). These results highlight the potential of DLL3-targeted PET imaging in selecting patients for targeted therapies and suggest promising therapeutic avenues in SCNC. Moreover, this is the only study that translates a novel radiopharmaceutical from a preclinical setting to the clinical one (although on a single patient from an ongoing trial). In the second study by Sharma et al. [25], six types of DLL3-targeted radioimmunoconjugates were examined in mouse xenograft models: for each conjugate, three mice were used for PET imaging and five for biodistribution analyses, covering a spectrum of small-cell tumours including NEPC. The study aimed to create and evaluate new ^89^Zr-labelled radiotracers against DLL3 for immuno-PET applications. The antibody used, SC16-MB1, was modified for site-specific radiolabelling to improve in vivo stability and performance. Site-specifically modified immunoconjugates of SC16-MB1 were studied using a maleimide-thiol linker (less stable) and a phenyloxadiazolyl methyl sulfone (PODS)-thiol linker (more stable and selective). Both tracers demonstrated high tumour uptake in DLL3-positive murine xenografts, with the PODS-based conjugate showing lower renal uptake, suggesting reduced potential toxicity and better image quality.

In the last paper, by Varuzhanyan et al. [26], large-scale analyses of organoids and xenografts derived from 10,529 patient tumours and 1466 human cancer cell lines were conducted, including PC cells; for this purpose, two tracers were tested: ^18^F-benzyltriphenylphosphonium (BnTP), targeting mitochondrial membrane potential, and ^18^F-FDG. The authors investigated mitochondrial metabolism’s role, driven by peroxisome proliferator-activated receptor gamma coactivator 1-alpha (PGC-1α), a master regulator protein that controls energy metabolism and mitochondrial biogenesis, in the progression and subtype determination of SCNC, focusing on the lineage marker achaete-scute homologue 1 (ASCL1) subtype, a basic helix-loop-helix transcription factor that plays a critical role in neuronal and neuroendocrine development. Using micro-PET/CT with ^18^F-FDG and the mitochondrial membrane potential tracer ^18^F-BnTP in xenograft models, they showed that PGC-1α overexpression enhances mitochondrial function and tumour aggressiveness. The study highlights PGC-1α as a metabolic driver of the ASCL1 SCNC subtype and suggests PET with ^18^F-BnTP as a promising tool to monitor mitochondrial activity and stratify patients.

Zhao et al. [27] additionally conducted a translational preclinical study proposing CUB Domain-Containing Protein 1 (CDCP1) as a novel target for radioligand therapy (RLT) in mCRPC, including PSMA-negative and SCPC cases. CDCP1, a transmembrane protein overexpressed in various aggressive tumours, was targeted using an anti-CDCP1 antibody (4A06) conjugated with ^89^Zr for PET imaging. In mouse models, ^89^Zr-4A06 PET detected CDCP1-positive tumours, including PSMA-negative SCNC models, demonstrating high uptake, favourable tumour-to-muscle and tumour-to-blood ratios, and superior uptake compared to ^68^Ga-PSMA-11 in PSMA-low tumours. The 4A06 antibody was also conjugated with Lutetium-177 (^177^Lu) for therapeutic use. In animal models of SCNC and adenocarcinoma, ^177^Lu-4A06 therapy significantly inhibited tumour growth, including in PSMA-negative tumours. This study provides robust preclinical evidence that CDCP1 is a promising diagnostic and therapeutic target, particularly in PSMA-refractory SCPC. PET imaging with ^89^Zr-4A06 may guide patient selection, while RLT with ^177^Lu-4A06 shows efficacy in animal models, supporting future clinical applications.

## 4. Discussion and Future Perspectives

This narrative review provides an overview on the potential role of PET/CT imaging in the diagnosis, characterization, and management of SCPC, a rare but clinically and prognostically relevant subgroup within broader PC populations with distinct biological features and clinical challenges. Although the available evidence remains limited and largely derived from retrospective studies including only small SCPC sub-cohorts, both clinical and preclinical studies underscore the peculiarity of SCPC within the broader spectrum of prostate cancer, highlighting both the potential and limitations of current molecular imaging in this challenging disease.

The characteristic dissociation between disease burden and PSA levels, with extensive metastatic disease despite low or undetectable PSA, limits the utility of PSA-based surveillance in SCPC patients. This phenomenon, well documented by Mahmoud et al. [22] who found that 21% of PC patients with radiographic progression at very low PSA levels showed small-cell or neuroendocrine features, emphasizes the need for PSA-independent imaging biomarkers in this tumour subgroup. Moreover, the propensity of SCPC to develop visceral and soft tissue metastases, rather than the bone-predominant pattern typical of adenocarcinoma, further complicates disease detection with conventional imaging modalities. From a nuclear medicine perspective, the molecular heterogeneity of SCPC, particularly the frequent loss of PSMA expression concurrent with neuroendocrine trans-differentiation, requires alternative or complementary imaging strategies. This review demonstrates that no single radiopharmaceutical can adequately characterize SCPC, supporting the need for a multimodal approach tailored to individual tumour biology.

Different radiotracers were employed across the included clinical studies, notably ^11^C-choline by Mahmoud et al. and Telo et al. [22,28], ^18^F-FDG by Shen et al. [23], [^68^Ga]Ga-DOTATATE by Bilen et al. [29], and 4A06-based conjugates with ^89^Zr/^177^Lu labelling by Zhao et al. [27].

Although clinical evidence remains limited and derived from mixed cohorts in which SCPC is often only a subgroup, with potential influence of selection bias and subgroup instability, ^18^F-FDG appears to be the most promising agent for SCPC imaging. The work by Shen et al. [23] provides compelling evidence for its diagnostic and prognostic utility, demonstrating significantly higher SUVmax values in SCPC compared to adenocarcinoma with neuroendocrine differentiation. They identified an optimal cut-off of 10.2 with excellent diagnostic accuracy, although this threshold was derived from a single study and therefore requires external validation. Beyond diagnosis, increased ^18^F-FDG uptake is also associated with poor survival, supporting its potential role in patients’ risk stratification and treatment planning. The biological basis for ^18^F-FDG avidity in SCPC reflects the metabolic reprogramming associated with neuroendocrine differentiation, characterized by high proliferative index, cellular dedifferentiation, and increased glycolytic metabolism, similarly to other small-cell neuroendocrine tumours. This metabolic signature may support both tumour detection and assessment of disease aggressiveness. However, several caveats must be acknowledged. First, ^18^F-FDG is not specific to SCPC and can be elevated in other aggressive prostate cancer subtypes, inflammatory processes, and concurrent malignancies. Second, the optimal timing of ^18^F-FDG PET/CT in the disease trajectory remains undefined and requires prospective validation, particularly to clarify whether its use should be limited to cases with clinical suspicion of neuroendocrine transformation or extended more broadly in high-risk mCRPC patients. Third, the metabolic heterogeneity within mixed tumours (containing both adenocarcinoma and small-cell components) may complicate interpretation and necessitate careful correlation with biopsy findings. Finally, variability in SUV measurements across scanners and acquisition/reconstruction protocols suggests that locally validated cut-offs are needed before clinical implementation.

**Figure 2:**
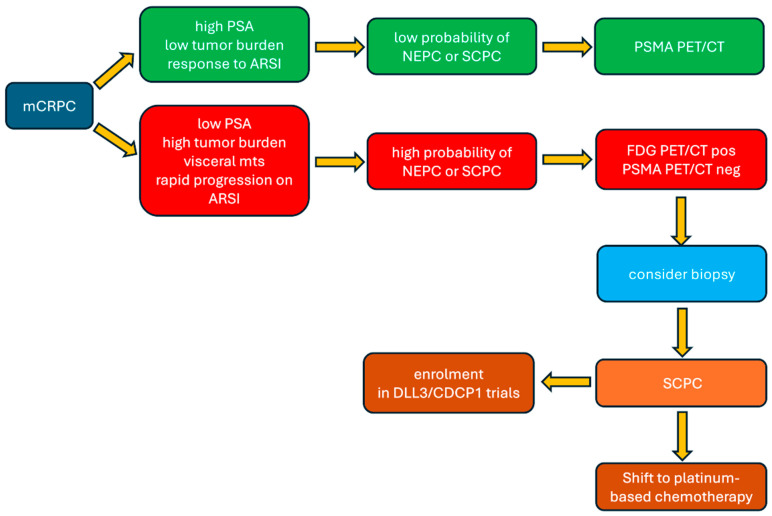
Flowchart of the suggested PET imaging and therapeutic pathway in SCPC, for clinicians’ purposes.

Regarding PSMA-targeted PET/CT, while it has revolutionized imaging of conventional prostate adenocarcinoma, this review confirms that it may fail to detect more aggressive disease such as SCPC due to the frequent loss of PSMA expression during neuroendocrine trans-differentiation. Similarly, ^11^C-choline PET/CT, while capable of detecting disease progression at low PSA levels, as shown by Mahmoud et al. [22], suffers from the same limitation: reduced uptake in dedifferentiated tumours. The observation by Telo et al. [28] that SCPC may explain false-negative ^11^C-choline scans in patients with biochemical recurrence underscores this critical limitation. From a clinical standpoint, this peculiar functional pattern may have important implications for patient management: ^18^F-FDG PET/CT should be considered for evaluating suspected neuroendocrine transformation in case PSMA or choline is negative or equivocal imaging despite clinical or biochemical progression, particularly when disease burden is disproportionate to PSA levels or following prolonged androgen receptor-targeted therapy. Taking all these considerations into account, several questions require urgent attention: when should ^18^F-FDG PET/CT be obtained in mCRPC patients, triggered by specific clinical features, or employed more broadly? How should discordant PSMA/FDG findings be interpreted and acted upon? Should high FDG uptake prompt histological confirmation before treatment modification? What is the role of the single-day dual-tracer protocol versus sequential imaging? Beyond these open questions, the integration of PET/CT findings into clinical decision-making algorithms requires validation. While elevated ^18^F-FDG uptake may suggest neuroendocrine differentiation, it does not automatically mandate specific therapy, particularly given the limited treatment options for SCPC. Rather, a positive FDG/negative PSMA imaging pattern could guide prompt confirmatory biopsy (when feasible) and leading clinicians to consider platinum-based chemotherapy versus continued androgen receptor-targeted therapy. An explicative flowchart of the suggested PET imaging and therapeutic pathway in SCPC is reported in Figure 2, for clinicians’ purposes. Finally, the role of somatostatin receptor (SSTR) imaging in SCPC, exemplified by ^68^Ga-DOTATATE PET/CT in the Bilen et al. study [29], represents an intriguing but incompletely understood avenue. While SSTR expression is well-established in various neuroendocrine tumours, its prevalence and intensity in SCPC remain variable and poorly characterized. Preliminary evidence of ^68^Ga-DOTATATE uptake in SCPC/NEPC cases suggests a promising diagnostic and potentially theranostic role in selected patients. However, the heterogeneous expression of SSTR in SCPC, both among patients and within different tumoral lesions in the same patient, suggests that SSTR imaging may be most valuable when integrated into a comprehensive imaging strategy rather than as a stand-alone modality. Moreover, the available data are limited to very small retrospective series, precluding definitive conclusions on SSTR-imaging value in SCPC patients and highlighting the need of prospective studies correlating SSTR expression patterns with histopathological features, clinical outcomes, and response to PRRT.

When considering the preclinical setting, investigations of DLL3-targeted imaging represent one of the most promising developments in SCPC molecular imaging. DLL3, a Notch pathway inhibitor overexpressed in aggressive neuroendocrine tumours including small-cell lung cancer and SCPC, offers exceptional specificity for this tumour subtype, while being largely absent in normal tissues and conventional adenocarcinoma. The works by Chou et al. [24] and Sharma et al. [25] demonstrate both the technical feasibility and biological rationale for DLL3-directed immuno-PET imaging with ^89^Zr-labelled tracers, showing high tumour-to-background ratios and the ability to quantify target expression, with potential therapeutic applications through antibody–drug conjugates or bispecific T-cell engagers. Preliminary clinical evidence of therapeutic efficacy with the T-cell engager AMG 757 in a DLL3-expressing SCPC case provides a proof of concept for this theranostic approach. From a nuclear medicine perspective, the site-specific PODS-based conjugation strategies employed by Sharma et al. [25], which demonstrated reduced renal uptake, exemplify the translational optimization required for clinical implementation, both in terms of reduced toxicity and better image quality. Moreover, the longer half-life of ^89^Zr (78.4 h) provides adequate imaging windows for antibody-based tracers while enabling serial imaging to monitor target expression dynamics during the treatment course. Beyond DLL3, Zhao et al. [27] investigated CDCP1 as an alternative target for radioligand therapy in PSMA-negative disease, expanding the theranostic arsenal. The anti-CDCP1 antibody ^89^Zr-4A06 demonstrated superior uptake compared to ^68^Ga-PSMA-11 in PSMA-negative SCPC, while ^177^Lu-4A06 showed therapeutic efficacy, suggesting a viable pathway for patients whose tumours lack conventional imaging targets. These findings underscore the importance of characterizing the molecular landscape of individual tumours to guide personalized imaging and therapeutic strategies.

Finally, the work by Varuzhanyan et al. [26] investigating mitochondrial metabolism through ^18^F-BnTP PET imaging introduces a fundamentally different biological dimension to SCPC imaging. The identification of PGC-1α-driven mitochondrial reprogramming as a driver of the ASCL1 SCPC subtype, and the ability to visualize this metabolically through mitochondrial membrane potential imaging, suggests exciting possibilities for both biological stratification and therapeutic targeting. If validated clinically, mitochondrial imaging could complement glycolytic imaging (^18^F-FDG) to provide a more comprehensive picture of tumour metabolism, potentially guiding targeted therapies, with dual-tracer protocols representing a feasible strategy for more complete tumour characterization.

Taking into account all these findings, the heterogeneity of SCPC, encompassing variability in PSMA expression, neuroendocrine marker expression, SSTR expression, glycolytic activity, and emerging targets, suggests that single-tracer strategies will prove insufficient for comprehensive disease characterization. Instead, the future likely involves adaptive, biology-driven imaging strategies in which initial comprehensive characterization (potentially by ^18^F-FDG, PSMA, and SSTR imaging) establishes the molecular profile of disease, followed by targeted surveillance and response assessment with the most appropriate tracer(s) for each patient.

Finally, several research priorities emerge from this review. First, prospective comparative studies of ^18^F-FDG versus PSMA tracers in patients with clinical or biochemical features suggesting neuroendocrine transformation are essential, ideally comparing dual-tracer protocols with same-day or sequential imaging and including correlation with histopathology, disease extent, and clinical outcomes. Second, rapid clinical translation of novel tracers targeting SCPC-specific antigens (particularly DLL3 and CDCP1) through phase I/II imaging trials is needed, also considering their theranostic potential. Third, radiomics and artificial intelligence applications to PET/CT imaging in PC should specifically address SCPC detection, with machine learning algorithms trained to identify imaging patterns predictive of neuroendocrine differentiation; integration of PET imaging features with clinical, genomic, and liquid biopsy data could yield powerful predictive models. Fourth, longitudinal imaging studies tracking the evolution from adenocarcinoma to SCPC could provide critical insights into transformation kinetics and early imaging biomarkers [30,31]; serial imaging during androgen receptor-targeted therapy in high-risk patients might reveal metabolic shifts preceding histological transformation. Finally, standardization of acquisition protocols, reconstruction parameters, and interpretation criteria for ^18^F-FDG PET/CT in prostate cancer, particularly for SCPC, is needed to enable multicentre collaboration and data pooling.

### Limitations

This narrative review must be interpreted in the context of several important limitations. First, the paucity of studies specifically focused on SCPC means that much of the evidence is derived from mixed populations, where SCPC only represents a minority: this aspect limits the statistical power and generalizability of our findings; moreover, case reports were not considered, since data derived from individual and often atypical findings lack generalizability and their results may be somehow inconsistent with those obtained from more complex population-based studies. Second, all clinical studies reviewed were retrospective, introducing potential selection bias and limiting causal inference. Third, the absence of head-to-head comparative studies prevents definitive conclusions regarding the relative performance of different tracers. Fourth, the predominance of single-centre studies limits external validity; additionally, the preclinical studies, while providing important biological insights and proof-of-concept data, may not fully recapitulate the complexity of SCPC in human subjects, particularly regarding tumour heterogeneity, immune microenvironment, and treatment effects. Moreover, the translation of promising preclinical imaging agents to clinical practice has historically proven challenging, with many agents failing due to unfavourable human pharmacokinetics, immunogenicity, or lack of clinical benefit despite good preclinical performance.

## 5. Conclusions

SCPC represents a unique challenge in molecular imaging, characterized by the need for metabolic and functional imaging approaches beyond conventional imaging. From our review on the diagnostic limitations of PSMA and choline-based imaging in this population, ^18^F-FDG PET/CT emerges as the best supported tool for SCPC detection, characterization, and prognostication.

In clinical practice, the optimal approach to SCPC imaging likely involves recognition of the disease’s biological heterogeneity and adaptation of imaging strategies accordingly: in patients with clinical features suggesting neuroendocrine transformation, particularly low PSA relative to disease burden, rapid progression despite androgen receptor-targeted therapy, or visceral metastases, ^18^F-FDG PET/CT should be recommended, even regardless of PSMA PET/CT findings. In addition, new emerging targets, particularly DLL3 and CDCP1 in preclinical settings, offer exciting theranostic possibilities that could impact on both diagnosis and treatment of this aggressive disease. So, the future integration of novel targeted agents into clinical practice promises to further refine our ability to detect, characterize, and ultimately treat this challenging disease.

In the future, the rarity of SCPC makes prospective clinical trials challenging and necessitates multicentre collaboration and innovative trial designs, so collaboration between nuclear medicine physicians, oncologists, pathologists, and basic scientists will be essential to translate imaging advances into improved patient outcomes: the ultimate measure of success will not simply be the better detection of SCPC, but rather the use of imaging-derived information to guide more effective, personalized therapeutic strategies that improve survival and quality of life for these patients.

## Figures and Tables

**Table 1:** Main characteristics of the articles included in the review.

Study	Year	Type	Design	Population/Models	SCPC/NEPC Representation	Radiopharmaceutical
Mahmoud et al. [22]	2024	Clinical	Retrospective; small SC-NEPC subgroup within larger cohort	1323 patients with rDP PCa on ^11^C-choline PET/CT; 220 with low PSA < 0.5 ng/mL	6 SC-NEPC at diagnosis (3%); 55 re-biopsied, 42 evaluable→9 SC-NEPC (21%)	^11^C-choline PET/CT
Shen et al. [23]	2023	Clinical	Retrospective; included well-characterized NEPC subset	44 NEPC patients	13 SCNC + 31 adenocarcinomas with NE differentiation	^18^F-FDG PET/CT
Zhao et al. [27]	2022	Clinical + Preclinical	Retrospective + preclinical validation; theranostic approach	119 mCRPC biopsies, 12 PDX models	SCNC + adenocarcinoma (exact distribution not specified)	^89^Zr-4A06 PET/CT; ^177^Lu-4A06
Telo et al. [28]	2020	Clinical	Large retrospective cohort; SCPC not isolated	5792 patients with BCR and high PSA	SCNC proportion not specified	^11^C-choline PET/CT
Bilen et al. [29]	2022	Clinical	Small retrospective series; targeted SSTR expression	16 patients with mCRPC	2 NEPC (12.5%)	[^68^Ga] Ga-DOTATATE PET/CT
Chou et al. [24]	2023	Preclinical (plus 1 patient from clinical trial)	Translational biopsy analysis; in vitro + in vivo	24 mCRPC biopsies (12 SCPC, 12 adenocarcinoma)	50% SCPC	DLL3-targeted tracers
Sharma et al. [25]	2021	Preclinical	In vivo PET imaging (3 mice/group) and biodistribution (5 mice/group)	Mouse xenografts; 6 DLL3-targeted radio-immunoconjugates	Included SCPC/NEPC models	DLL3-targeted tracers
Varuzhanyan et al. [26]	2024	Preclinical	Translational study across multiple tumour types	10,529 tumours/1466 cell lines incl. PCa organoids and xenografts	Included prostate cancer but not exclusive to SCPC	^18^F-BnTP, ^18^F-FDG

SC-NEPC (small cell-neuroendocrine prostate cancer); rDP (radiographic disease progression); PCa (prostate cancer); PET/CT (positron emission tomography/computed tomography); NE (neuroendocrine); FDG (fluorodeoxyglucose); mCRPC (metastatic castration-resistant prostate cancer); PDX models (patient-derived xenograft); SCNC (small-cell neuroendocrine carcinoma); SCPC (small-cell prostate cancer); BCR (biochemical recurrence); DLL3 (delta-like ligand 3); BnTP (benzyltriphenylphosphonium).

**Table 2:** Main PET/CT technical characteristics in clinical studies.

Study	Radiopharmaceutical	Injected Activity	Scan Delay	Field of View	PET/CT Analysis	PET/CT Reconstruction
Mahmoud et al. [22]	^11^C-choline	n.r.	5 min	From orbits to tights	SQA (SUVmax)	n.r.
Shen et al. [23]	^18^F-FDG	2.5–4 MBq/kg	60 min + 140 min for delayed PET imaging of the pelvis	From base of skull to upper tights	SQA (SUVmax)	n.r.
Telo et al. [28]	^11^C-choline	370–555 MBq	3–5 min	From base of the skull to mid-tights	n.r.	n.r.
Bilen et al. [29]	^68^GaDOTATATE	200 ± 11 MBq	55–70 min	From base of the skull to upper tights	SQA (SUVmax, SUVmean)	3 iterations/24 subsets, 6.4 mm filter cut-off, 3.75 mm slice thickness

PET/CT (positron emission tomography/computed tomography); n.r. (not reported); FDG (fluorodeoxyglucose); SQA (semiquantitative analysis).

## Data Availability

No new data were created or analyzed in this study.

## Abbreviations

The following abbreviations are used in this manuscript:

SCPC	Small-Cell Prostate Cancer
mCRPC	metastatic Castration-Resistant Prostate Cancer
AR	Androgen Receptor
PET/CT	Positron Emission Tomography/Computed Tomography
PSMA	Prostate Specific Membrane Antigen
^18^F-FDG	Fluorine-18 fluorodeoxyglucose
NEPC	Neuro-Endocrine Prostate Cancer
rDP	radiographic Disease Progression
SC-NEPC	Small-Cell Neuro-Endocrine Prostate Cancer
Ad-NED	Adenocarcinoma with Neuro-Endocrine Differentiation
SUVmax	Maximum Standardized Uptake value
PDXs	Patient-Derived Xenografts
BCR	Biochemical Recurrence
DLL3	Delta-Like Ligand 3
scFv	single-chain variable fragment
PODS	Phenyloxadiazolyl methyl Sulfone
BnTP	^18^F-benzyltriphenylphosphonium
PGC-1α	Proliferator-activated receptor Gamma Coactivator 1-alpha
ASCL1	Achaete-scute homologue 1
CDCP1	CUB Domain-Containing Protein 1
RLT	Radioligand Therapy
^177^Lu	Lutetium-177
